# Anterior chamber flare and central macular thickness after trabeculectomy versus after phacoemulsification

**DOI:** 10.1111/aos.70032

**Published:** 2025-11-19

**Authors:** Yasmeen Ahmed, Jesper Høiberg Erichsen, Afrouz Ahmadzadeh, Lars Morten Holm, Line Kessel, Daniella Bach‐Holm

**Affiliations:** ^1^ Department of Ophthalmology Copenhagen University Hospital—Rigshospitalet Copenhagen Denmark; ^2^ Department of Clinical Medicine University of Copenhagen Copenhagen Denmark

**Keywords:** anterior chamber flare, cataract surgery, central macular thickness, glaucoma surgery, inflammation, phacoemulsification, trabeculectomy

## Abstract

**Purpose:**

To compare the inflammatory response in the eye after trabeculectomy to after phacoemulsification, focusing on anterior chamber flare (AC flare) and central macular thickness (CMT).

**Methods:**

Data from 436 participants in two randomized controlled trials were analysed. Anterior chamber flare was measured in 69 participants undergoing trabeculectomy and 367 participants undergoing phacoemulsification preoperatively. Postoperative assessments were made on day 7 in trabeculectomy participants and on day 3 in phacoemulsification participants. CMT was assessed at baseline and 3 months postoperatively in both groups and 4 weeks postoperatively for trabeculectomy participants and 3 weeks postoperatively for phacoemulsification participants.

**Results:**

At baseline, AC flare and CMT were comparable between the groups. Early postoperatively, AC flare was 22.1 ph/ms [95% CI 19.1, 25.7] for trabeculectomy and 18.9 ph/ms [95% CI 17.8, 20.2] for phacoemulsification. Both groups significantly increased in AC flare from their baseline values, but the difference in increase between them was not significant (*p* = 0.46). In the trabeculectomy group, CMT showed no significant increase at 4 weeks but a significant rise of 2.3 microns from baseline to 3 months from 241 microns [95% CI 235.9, 246.1] to 243.3 microns [95% CI 237.6, 249.1] (*p* = 0.038). In the phacoemulsification group, CMT was significantly increased at 3 weeks and remained significantly elevated at 3 months from 242.6 microns [95% CI 240.4, 244.8] to 249.8 microns [95% CI 247.3, 252.4], increased by 7.2 microns (*p* < 0.0001). CMT increased significantly more after phacoemulsification compared to trabeculectomy at 3 to 4 weeks (*p* = 0.014) and 3 months (*p* < 0.0001) with respect to baseline values.

**Conclusion:**

No significant difference in AC flare was found between trabeculectomy and phacoemulsification participants at baseline and early postoperatively. CMT did not increase at 4 weeks but increased significantly at 3 months after trabeculectomy. Phacoemulsification led to a significantly higher CMT increase at both 3 weeks and 3 months with respect to baseline compared to trabeculectomy.

## 1 INTRODUCTION

Glaucoma and cataract are the primary causes of, respectively, irreversible and reversible vision loss globally (GBD 2019 Blindness and Vision Impairment Collaborators & Vision Loss Expert Group of the Global Burden of Disease Study 2021). Glaucoma is an optic neuropathy characterized by progressive degeneration of retinal ganglion cells and optic nerve fibres, loss of visual field, and ultimately blindness. Lowering intraocular pressure (IOP) is the only known treatment to slow glaucoma progression. Therefore, if there is an inadequate IOP lowering effect of pressure‐reducing eye drops, IOP lowering procedures, such as laser trabeculoplasty and filtering surgery, may be necessary (Jayaram et al., 2023; Rao & Cruz, 2022). Cataract, characterized by opacities in the lens, can be cured by surgery, where the natural lens is replaced with an artificial intraocular lens (IOL) (Asbell et al., 2005).

Assessing inflammatory markers, such as anterior chamber flare (AC flare) and central macular thickness (CMT), is important for monitoring post‐surgical inflammation. AC flare occurs due to the release of inflammatory mediators that compromise the blood–aqueous barrier, leading to an increased protein concentration in the aqueous humour. This condition is characterized by enhanced protein reflection, giving the aqueous humour a cloudy or milky appearance when light is directed at it (Kesim et al., 2022; Tugal‐Tutkun & Herbort, 2010). Another marker of inflammation in the eye is CMT, which is an important measure for assessing macular oedema. The integrity of the blood–retinal barrier is crucial for maintaining retinal homeostasis. Inflammation in the eye can compromise the blood–retinal barrier due to the upregulation of inflammatory cytokines such as VEGF, TNF‐alpha, interleukins (e.g., IL‐6) and chemokines (Ozaki et al., 2014). These mediators promote vascular permeability and leukocyte infiltration, leading to fluid accumulation in the macula. This leakage of fluid and subsequent thickening of the macula is measured as an increase in CMT (Jang et al., 2020). An increased CMT may indicate conditions such as cystoid macular oedema or other retinal changes which can result in significant visual reduction (Verma et al., 2020).

Increased AC flare can occur after a trabeculectomy, where the blood–aqueous barrier is disrupted by the surgical trauma. The procedure directly affects the eye's tissues, including the iris, which is crucial in maintaining the barrier. This induces inflammation, releasing mediators that further compromise blood vessel permeability (Arimura et al., 2021). Reports indicate that postoperative increases in CMT and the development of cystoid macular oedema can occur after trabeculectomy due to the previously mentioned changes (Manabe et al., 2020). Administration of anti‐inflammatory treatment is essential to reduce postoperative inflammation, including minimizing fibrosis in the surgically created filtration bleb (Ahmadzadeh et al., 2023).

During cataract surgery lens proteins are released, mediating an inflammatory response that degrades the blood–ocular barrier and releases leukocytes (Flach, 1998). This leads to an increased AC flare and can lead to pseudo‐phakic cystoid macular oedema (PCME), a condition marked by the accumulation of fluid in the macula that may reduce visual function (Flach, 1998; Siriwardena et al., 2000). It has been reported that the peak incidence of PCME typically occurs around 3–4 weeks after surgery (Guo et al., 2015). To reduce these postoperative complications, including the prevention of uveitis, synechiae, and postoperative pain, anti‐inflammatory eyedrops are commonly administered (Samanta & Saraf, 2004).

The aim of this study was to investigate and compare inflammation in the eye after trabeculectomy and phacoemulsification using measures of AC flare and CMT. Understanding these inflammatory responses is crucial for optimizing postoperative management and visual outcomes in patients undergoing these procedures. Comparison of CMT following trabeculectomy and phacoemulsification is an area that has not yet been described in existing literature in contrast to comparison of AC flare (Siriwardena et al., 2000).

## 2 METHODS

### 2.1 Study design

This study is a comparative study of two randomized controlled trials, with one trial including participants who underwent trabeculectomy and the other trial including participants who underwent phacoemulsification. Participants could not be fully masked to treatment status due to the study design involving mono or combination therapy of steroidal and non‐steroidal anti‐inflammatory eyedrops. However, masking was maintained by blinding primary outcome assessors to randomization status and conducting all statistical analyses in a blinded manner. Both trials were conducted in accordance with the Declaration of Helsinki and were conducted in accordance with good clinical practice (GCP) guidelines and monitored by the GCP unit at Copenhagen University Hospital. The trials are registered on www.clinicaltrials.gov under the identifiers NCT04054830 for the trabeculectomy trial and NCT03383328 for the phacoemulsification trial (Ahmadzadeh et al., 2023; Erichsen et al., 2021). In cases where both eyes underwent surgery, only one eye per participant was included in the study through a computerized coin toss. All recruited participants were operated at The Eye Department, Rigshospitalet‐Glostrup, Denmark.

### 2.2 Participants

Participants who underwent trabeculectomy were included from August 2019 to July 2021 in the SNAP study (Steroids and/or Non‐steroidal Anti‐inflammatory drugs in the Postoperative regime after trabeculectomy). Inclusion criteria were patients over the age of 50 needing IOP‐lowering surgery due to primary open‐angle glaucoma (POAG), pseudo‐exfoliation glaucoma (PEXG), pigment dispersion glaucoma (PDG) or ocular hypertension (OHT). Exclusion criteria were angle‐closure glaucoma, neovascular glaucoma, traumatic glaucoma, intraocular surgery other than phacoemulsification and known steroid responders (Ahmadzadeh et al., 2023). The cataract group included patients with age‐related cataract from February 2018 to October 2019 in the SOAP study (Study for Optimizing Anti‐inflammatory Prophylaxis). Exclusion criteria in this group were other co‐existing eye diseases such as AMD, glaucoma, uveitis, DM and retinal diseases (Erichsen et al., 2021).

In both groups, women were required to be post‐menopausal. Participants from both groups received different types of anti‐inflammatory eyedrops postoperatively, as they were part of two separate studies comparing steroidal and nonsteroidal anti‐inflammatory regimens. The postoperative anti‐inflammatory regimes were as follows for the included participants:

### 2.3 Trabeculectomy group

- Combination therapy with NSAID (Voltaren Ophtha 1 mg/mL, GSK Consumer Healthcare) and steroid (Monopex 1 mg/mL, Théa).
- Monotherapy with one of the above mentioned eyedrops.

### 2.4 Phacoemulsification group

- Combination therapy with prednisolone (Pred Forte 1%, prednisolone acetate, Allergan) and ketorolac (Acular 0.5%, Allergan).
- Monotherapy with ketorolac (Acular 0.5%, Allergan).

We were able to pool and further analyse the data from both groups because no significant differences were observed in AC flare and CMT across the various inflammatory treatment regimens (Ahmadzadeh et al., 2023; Erichsen et al., 2021).

### 2.5 Surgical technique

The trabeculectomy procedure involved creating a limbus‐based scleral flap and applying the antifibrotic drug mitomycin C subconjunctivally. Following this, a sclerostomy was made to remove a block of corneal and scleral tissue at the level of the trabecular meshwork, along with a peripheral iridectomy. The flap was then sutured to restrict the outflow of fluid. The procedure was concluded by injecting cefuroxime into the anterior chamber and dexamethasone subconjunctivally (Ahmadzadeh et al., 2023).

During phacoemulsification a main incision of 2.4 mm and a side port of 1 mm were created. Ultrasonic phacoemulsification was performed by which the lens was removed, followed by irrigation and aspiration of cortical material, and then implantation of an artificial intraocular lens. Cefuroxime was injected into the anterior chamber to conclude the procedure (Erichsen et al., 2021).

Both procedures were performed by experienced surgeons under local anaesthesia, except for one participant in the trabeculectomy group who underwent general anaesthesia (Ahmadzadeh et al., 2023; Erichsen et al., 2021).

### 2.6 Clinical assessments and outcomes

The primary outcomes of this study were AC flare and CMT. AC flare was measured using a laser flare photometer (KOWA FM‐600, Nagoya, Japan). Protein density in the anterior chamber was measured on non‐dilated eyes in a dark room and using the average of five measures in each eye. Measurements were performed by experienced clinicians and research technicians. Participants who underwent trabeculectomy and phacoemulsification had AC flare measured at baseline and day 7 and day 3 postoperatively, respectively. CMT was measured by optical coherence tomography (OCT). In the trabeculectomy study, a Spectral Domain OCT (Spectralis, Heidelberg) with scanning protocols of 8.8 mm by 8.8 mm, 768 × 496 b‐scans, was used, while in the phacoemulsification study, a Swept Source OCT (DRI OCT Triton, Topcon) with a ‘3D Macula’ scan of 7.0 mm by 7.0 mm, 512 × 256 b‐scans, was used. CMT was calculated by the built‐in software and reported as the central 1 mm subfield of the ETDRS grid. CMT was measured at baseline in both studies and postoperative measures were done after 4 weeks and 3 months in the trabeculectomy study and at 3 weeks and 3 months in the phacoemulsification study. Our SD‐OCT data were converted to SS‐OCT before comparison using the following conversion formula: SD‐OCT = 33.53 + 0.994 × SS‐OCT (Xiong et al., 2021).

### 2.7 Statistics

Statistical analyses were performed using the statistical software R, v 4.1.0 in RStudio. AC flare and CMT were analysed using a linear mixed model with an unstructured covariance pattern. The model handled missing values by maximum likelihood estimation based on a ‘missing at random’ assumption. AC flare measurements were logarithmically transformed using log2. A *p*‐value of <0.05 was considered statistically significant. *p*‐values were not adjusted for multiple testing.

AC flare was assessed at baseline and compared to 7 days after trabeculectomy versus 3 days after phacoemulsification, representing the early postoperative period. CMT was compared at baseline, at 4 weeks post‐trabeculectomy versus 3 weeks post‐phacoemulsification, and both groups were compared again at 3 months postoperatively.

Logarithmic transformation was chosen to assume normal distribution of data and to reduce the influence of outliers. All flare values were transformed using log2 and that changes in flare were to be interpreted as a fraction after back transformation from logarithmic scale.

*p*‐values were not adjusted for multiple testing, as the analyses were exploratory and defined post‐hoc. Given the limitations of available correction methods, adjusted *p*‐values were not presented, but results should be interpreted with caution.

Confidence intervals are presented as mean [95% CI] for CMT and median [95% CI] for AC flare after statistical analysis.

## 3 RESULTS

We included a total of 436 participants in the study of which 69 had a trabeculectomy and 367 had phacoemulsification performed. Baseline characteristics are shown in Table 1.

**TABLE 1:** Baseline characteristics.

Participants	Trabeculectomy (*n* = 69)	Phacoemulsification (*n* = 367)	*p*‐value
Female	30 (43)	236 (64)	0.002
Age, mean (SD)	71.3 (9.0)	72.3 (7.0)	0.26
Glaucoma diagnoses			
HTG	55 (79.9)		
PEXG	6 (8.7)		
NTG	5 (7.2)		
PDG	2 (2.9)		
OHT	1 (1.4)		
Phakia	46 (67)		
Pseudophakia	23 (33)		

*Note*: Values are *n* (%) unless otherwise indicated.

Abbreviations: HTG, high tension glaucoma; NTG, normal tension glaucoma; OHT, ocular hypertension; PDG, pigment dispersion glaucoma; PEXG, pseudo‐exfoliation glaucoma.

### 3.1 Baseline characteristics

There was a significantly higher proportion of women in the phacoemulsification group than in the trabeculectomy group (*p* = 0.002). Men had significantly higher baseline flare than women with a factor of 1.11 [95% CI 1.02, 1.22] (*p* = 0.02), but sex did not affect the change in flare from baseline to postoperative (*p* = 0.58). There was no difference in age between the two groups (*p* = 0.26). See Tables 1 and 2.

**TABLE 2:** Results on anterior chamber flare and central macular thickness.

	Trabeculectomy	Phacoemulsification
AC flare (ph/ms)^a^, median [95% CI]		
Baseline	11.1 [10.0, 12.5]	10.1 [9.6, 10.6]
7/3 days postoperatively^b^	22.1 [19.1, 25.7]^d^	18.9 [17.8, 20.2]^d^
CMT (microns), mean [95% CI]		
Baseline	241 [235.9, 246.1]	242.6 [240.4, 244.8]
4/3 weeks postoperatively^c^	242.9 [236, 249.9]	249.8 [246.7, 252.8]^d^
3 months postoperatively	243.3 [237.6, 249.1]^d^	249.8 [247.3, 252.4]^d^

Abbreviations: AC flare, anterior chamber flare; CMT, central macular thickness.

^a^ ph/ms = photon counts per millisecond.

^b^ 7 days post‐trabeculectomy and 3 days post‐phacoemulsification.

^c^ 4 weeks post‐trabeculectomy and 3 weeks post‐phacoemulsification.

^d^ Indicates statistically significant increase from baseline values.

### 3.2 Anterior chamber flare

Trabeculectomy participants had a baseline median AC flare of 11.1 ph/ms [95% CI 10.0, 12.5], which increased to 22.1 ph/ms [95% CI 19.1, 25.7] 7 days post‐surgery, representing a 1.99‐fold increase [95% CI 1.71, 2.31]. In the phacoemulsification group, the baseline median AC flare was 10.1 ph/ms [95% CI 9.6, 10.6], increasing to 18.9 ph/ms [95% CI 17.8, 20.2] 3 days after surgery, corresponding to a 1.87‐fold increase [95% CI 1.75, 1.99]. The increases from baseline to post‐surgery were statistically significant in both groups (*p* < 0.0001).

**FIGURE 1:**
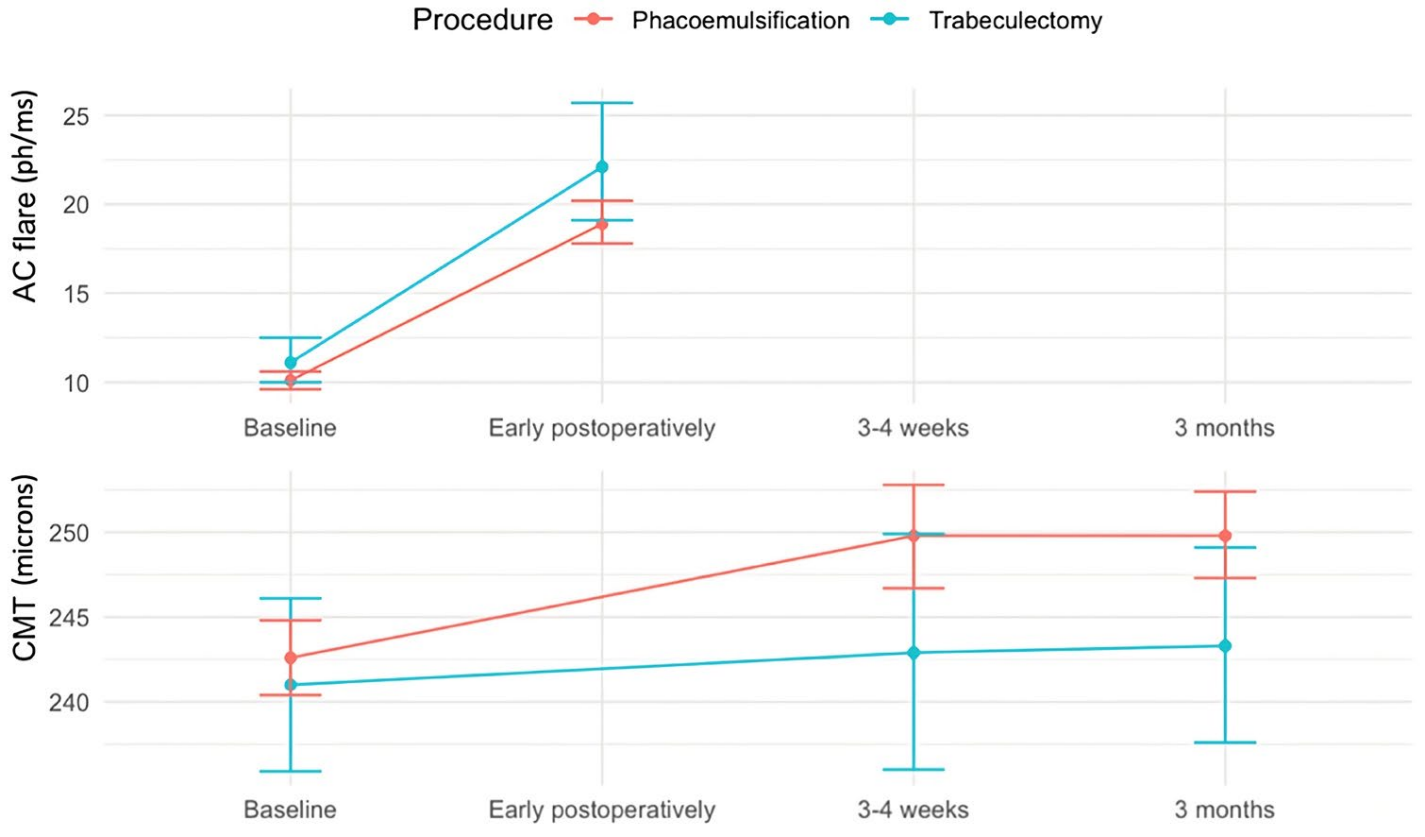
Changes in anterior chamber flare and central macular thickness over time. Bars indicate 95% confidence intervals. 3–4 weeks, 3 weeks after cataract surgery and 4 weeks after trabeculectomy; AC flare, anterior chamber flare; CMT, central macular thickness; Early postoperatively, postoperative control 7 days after surgery in patients with trabeculectomy and 3 days after surgery in patients with phacoemulsification surgery; ph/ms, photon counts per millisecond.

At baseline, trabeculectomy participants had a 1.10‐fold higher AC flare [95% CI 0.97, 1.24] compared to phacoemulsification participants; however, this difference was not statistically significant. The difference in the increase in AC flare between the two groups was also not significant (*p* = 0.46) (see Table 2 and Figure 1).

### 3.3 Central macular thickness

At baseline, mean CMT was 241.0 microns [95% CI 235.9, 246.1] in the trabeculectomy group and 242.6 microns [95% CI 240.4, 244.8] in the phacoemulsification group (*p* = 0.57). In the trabeculectomy group, mean CMT increased to 242.9 microns [95% CI 236.0, 249.9] 4 weeks postoperatively (*p* = 0.32). At 3 months postoperatively, mean CMT further increased to 243.3 microns [95% CI 237.6, 249.1], which was significantly increased compared to baseline (*p* = 0.038). Phacoemulsification participants increased to 249.8 microns [95% CI 246.7, 252.8] 3 weeks after surgery and remained increased at 249.8 microns [95% CI 247.3, 252.4] 3 months postoperatively, with both increases being statistically significant compared to baseline (*p* < 0.0001).

Mean CMT increased significantly more after phacoemulsification compared to trabeculectomy at both 3 to 4 weeks and 3 months after surgery with respect to baseline (*p* = 0.014 and *p* < 0.0001, respectively) (see Table 2 and Figure 1).

### 3.4 Retinal changes on OCT

Postoperative OCT scans revealed retinal changes following both procedures. Post‐trabeculectomy, 2 out of 69 participants (2.9%) had developed pigment epithelium detachment and another two participants (2.9%) developed peripapillary retinal oedema with fluid accumulation in the inner and outer nuclear layers. In the phacoemulsification group 9 out of 367 participants (2.45%) developed pseudo‐phakic macular oedema defined by the detection of cystoid abnormalities on OCT (Flach, 1998).

### 3.5 Subanalysis of pseudo‐exfoliation's effect on AC flare

Subanalysis of the effect of pseudo‐exfoliation on AC flare showed no significant impact on either surgical group. See Table 3.

**TABLE 3:** Flare increase factor of PEX/PEXG on AC flare increase in, respectively, phacoemulsification and trabeculectomy group.

	Flare increase factor [95% CI]	*p*‐value
PEX in phacoemulsification group	1.01 [0.82, 1.24]	0.95
PEXG in trabeculectomy group	1.11 [0.77, 1.60]	0.58

Abbreviations: AC flare, anterior chamber flare; PEX, pseudo‐exfoliation; PEXG, pseudo‐exfoliation glaucoma.

## 4 DISCUSSION

This comparative study of two randomized controlled trials aimed to investigate the differences in AC flare and CMT following trabeculectomy and phacoemulsification. While both procedures did cause a significant increase in AC flare early postoperatively, we did not find any statistical difference in AC flare when comparing the two procedures. After trabeculectomy CMT did not increase after 4 weeks but there was a small significant increase at 3 months. CMT increased significantly after 3 weeks and 3 months in the phacoemulsification group. CMT showed a significantly greater increase after phacoemulsification compared to trabeculectomy at both 3 to 4 weeks and 3 months after surgery compared to baseline.

Our results showed no significant difference in AC flare between participants undergoing trabeculectomy and those undergoing phacoemulsification at baseline and early postoperative periods. This suggests that both procedures induce a similar level of inflammation in the anterior chamber initially. It has been hypothesized that cataract surgery may cause a higher increase in AC flare compared to other intraocular procedures due to a large volume of fluid passing through the eye during surgery and the release of lens crystallins and lens epithelial cells into the aqueous humour. These factors may increase the production of fibrogenic cytokines in the aqueous humour of participants undergoing phacoemulsification (Siriwardena et al., 2000). Research has shown that the fibrogenic cytokine monocyte chemotactic protein‐1 is elevated after phacoemulsification (Kawai et al., 2012). The SOAP study found no correlation between AC flare and cumulative dissipated energy used during ultrasound (Erichsen et al., 2021). In a prior study, AC flare was significantly greater in phacoemulsification participants after 6 weeks and 3 months compared to trabeculectomy. It found AC flare to return to baseline in the trabeculectomy group by week 4, but not until 6 months post‐phacoemulsification. Both groups received topical steroids; in the trabeculectomy group 4–8 times daily for 6 weeks and in the phacoemulsification group 4–6 times daily for at least 4 weeks (Siriwardena et al., 2000). The reason we do not observe a significant difference in AC flare between the two procedures, unlike Siriwardena et al., could be due to their exclusion of pseudo‐exfoliation glaucoma (PEXG). In our study, 8.7% of the trabeculectomy group had PEXG. A study of 96 eyes with POAG and PEXG found that AC flare significantly increased after trabeculectomy only at 1 week relative to baseline, but not at later time points from 2 weeks onwards. However, they observed a significantly higher AC flare in participants with PEXG compared to POAG at various time points, including 1 week postoperatively, where the mean flare in the PEXG group was 87.9 ph/ms [95% CI 63.5–112.2], whereas in the POAG group, it was 38.6 ph/ms [95% CI 22.0–55.2] (Tanito et al., 2021). The SNAP study, which included POAG and PEXG participants, also found that AC flare was increased at 1 day and 1 week postoperatively but was not significantly increased from 2 weeks onwards (Ahmadzadeh et al., 2023). Our sub‐analysis found that neither PEX nor PEXG significantly influenced postoperative AC flare in the respective groups.

Based on our analysis, CMT did not increase 4 weeks post‐trabeculectomy, but there was a small yet significant increase after 3 months. Current literature has reported varying findings on CMT post‐trabeculectomy. One study of 64 phakic glaucoma participants found no statistically significant increase at 1 and 3 months (Nilforushan et al., 2022), while another study of 106 eyes with glaucoma observed a significant increase in CMT 1 week after surgery, with additionally the nasal and central subfields significantly increased after 3 months. Also, this study found a positive correlation of CMT increase and IOP reduction (Kadziauskienė et al., 2017). It is suggested that thickening of the macula after trabeculectomy could be a natural response to retinal stress caused by a sudden drop in IOP. This may occur due to an imbalance between capillary pressure and interstitial fluid pressure (Pitale et al., 2016). A similar correlation was observed between IOP and retinal nerve fibre layer increase post‐trabeculectomy (Aydin et al., 2003).

According to our results, CMT increased significantly at both 3 weeks and 3 months post‐phacoemulsification, remaining consistently elevated by 7.2 microns from baseline at both time points. This stable and relatively mild increase in macular thickness after phacoemulsification may be due to both subclinical changes, without impact on visual acuity, as well as the impact of changes in media opacity caused by a clearer lens on the measurement technique (Jagow et al., 2007). The preoperative image quality is reduced because cataract leads to decreased signal strength on OCT scans, which may explain why postoperative macular thickness is slightly increased (Mwanza et al., 2011; Van Velthoven et al., 2006). This is also suggested by another study, as they observed a significant correlation between nuclear density in the crystalline lens and postoperative increase in retinal thickness. They also found that both the cumulative dissipated energy used and the ultrasound time correlated significantly with the increase in retinal thickness (Mackenbrock et al., 2023). Prior studies find differing changes in CMT after phacoemulsification. A study of 65 participants showed that changes in CMT after 1, 3 and 6 months were statistically insignificant, and the subclinical increase returned to baseline at 6 months postoperatively (Yilmaz et al., 2016). However, in another study of 40 participants, CMT significantly increased from the first month after surgery, peaking at 2 months. By 6 months, CMT decreased with no significant difference compared to baseline, suggesting changes are only temporary due to inflammation (Gharbiya et al., 2013). Both Yilmaz et al. and Gharbiya et al. operated on eyes with senile cataract, and no other ocular diseases, and reported CMT as the 1 mm central subfield scan using Spectral Domain OCT (Gharbiya et al., 2013; Yilmaz et al., 2016).

Regarding retinal changes that may be expressed as an increased CMT, we did not observe any cases of cystoid macular oedema (CME) in our trabeculectomy group while we observed PCME in 2.45% participants post‐phacoemulsification. Previous studies have reported an incidence of CME ranging from 3% to 9% following trabeculectomy (Gietzelt et al., 2024) and an incidence range of approximately 4% to 20% following cataract surgery (Manabe et al., 2020). We also observed retinal changes post‐trabeculectomy, including pigment epithelial detachment and peripapillary retinal oedema. These retinal changes result from a compromised blood–retinal barrier, causing protein and fluid to leak into the retina (Kohli et al., 2024). Postoperative hypotony may contribute to these retinal changes, as a sudden drop in intraocular pressure can lead to an imbalance between capillary pressure and interstitial fluid pressure (Pitale et al., 2016).

To the best of our knowledge, no other study has compared inflammation in the eye after trabeculectomy and phacoemulsification using both AC flare and CMT, and no study has compared CMT after both procedures. One of the strengths of this study is its relatively large population when assessing CMT in trabeculectomy participants. This was achieved by pooling data from the participants in the SNAP study (Ahmadzadeh et al., 2023). We also had a long follow‐up duration of CMT of 3 months, allowing us to investigate the development of inflammation in the posterior part of the eye. We are not aware of any other studies comparing AC flare following different postoperative anti‐inflammatory regimens after trabeculectomy, apart from the study from which we pooled data for this study (Ahmadzadeh et al., 2023). On the other hand, several studies have measured anterior chamber flare following phacoemulsification. Of these, three found significant differences between anti‐inflammatory regimens on AC flare levels: two favoured steroids over NSAIDs (Malik et al., 2016; Trinavarat et al., 2003) and one favoured NSAIDs over steroids (Coassin et al., 2019). The study included in this research reported no significant difference between monotherapy with NSAID versus steroids and NSAID in combination (Erichsen et al., 2021). Overall, both drug classes show clinical value.

Given that central macular thickness can be affected by other retinal conditions, this was minimized by excluding patients with exudative AMD in the phacoemulsification group, as exudative AMD can independently influence central macular thickness. The exclusion was therefore related more to the analysis of retinal outcomes than to anterior chamber flare levels specifically. Although exudative AMD was not an explicit exclusion criterion in the trabeculectomy group, no included participants had this condition, thereby reducing the risk of confounding in the combined analysis.

Our study has potential limitations. First, we used two different OCT machines: Spectral Domain OCT (Spectralis, Heidelberg) and Swept‐Source OCT (DRI OCT Triton, Topcon), which could introduce variability in our measurements. Current literature has shown that macular and retinal thickness measured with Spectral Domain OCT (SD‐OCT) is significantly higher compared to Swept Source OCT (SS‐OCT) (Bahrami et al., 2017; Nam et al., 2024; Tan et al., 2015). Due to this, all our SD‐OCT data was converted to SS‐OCT before comparison using the following conversion formula: SD‐OCT = 33.53 + 0.994 × SS‐OCT (Xiong et al., 2021). This conversion is supported by Xiong et al. who reported a high level of agreement between SD‐OCT and SS‐OCT measurements (Intraclass Correlation Coefficient (ICC) > 0.866), classifying them as ‘almost perfect’ (ICC = 0.81–1.0) (Xiong et al., 2021). Second, there was a variation in the follow‐up days between the two participant groups, and our AC flare data were limited to 7 days postoperatively for trabeculectomy and 3 days postoperatively for phacoemulsification. This difference may confound comparisons of early postoperative inflammation, as peak inflammatory responses could occur at different time points. Moreover, the sample size of the trabeculectomy group was considerably smaller than that of the phacoemulsification group, which limits the statistical power and may reduce the reliability or generalizability of conclusions drawn from between‐group comparisons. These limitations should be kept in mind when interpreting the findings. Our statistical analysis was post hoc, meaning the patterns we explored were not pre‐specified in the original research protocol. As the analyses are exploratory and no adjustments for multiple comparisons were applied, the findings should be interpreted with caution. We have therefore been careful in drawing conclusions from borderline significant results to minimize the risk of random findings.

Future studies should explore the use of CMT as a measure of inflammation post‐trabeculectomy and its effect on surgical outcomes. It is also relevant to study the incidence and severity of macular oedema, including cystoid macular oedema and other retinal changes after trabeculectomy, to better understand the risk factors and improve management strategies.

This study shows that after phacoemulsification CMT increases more and for a longer time compared to after trabeculectomy as a possible sign of an increased and sustained inflammatory response. The clinical significance of this study is that in patients with postoperative visual changes that are either unexpected or persist beyond the immediate postoperative phase, it may be relevant to consider OCT to evaluate for possible macular changes.

## 5 CONCLUSION

Our study demonstrated comparable levels of AC flare between participants who underwent trabeculectomy and phacoemulsification at baseline and early postoperatively, while both groups of participants did increase significantly from baseline. Post‐trabeculectomy there was no increase in CMT after 4 weeks and only a marginally significant increase in CMT at 3 months compared to baseline. Phacoemulsification led to a significantly greater increase in CMT after 3 weeks and it remained significantly and consistently elevated after 3 months. Comparing the two procedures, phacoemulsification resulted in a significantly greater increase in CMT compared to trabeculectomy at both 3 to 4 weeks and 3 months postoperatively relative to baseline.

## References

[aos70032-bib-0001] Ahmadzadeh, A. , Schmidt, B.S. , Bach‐Holm, D. & Kessel, L. (2023) Early inflammation control after trabeculectomy by steroid and non‐steroidal eye drops: a randomized controlled trial. Ophthalmology and Therapy, 12, 969–984. 36602718 10.1007/s40123-022-00636-2 PMC10011236

[aos70032-bib-0002] Arimura, S. , Iwasaki, K. , Orii, Y. , Takamura, Y. & Inatani, M. (2021) Comparison of 5‐year outcomes between trabeculectomy combined with phacoemulsification and trabeculectomy followed by phacoemulsification: a retrospective cohort study. BMC Ophthalmology, 21, 1–8. 33894759 10.1186/s12886-021-01949-9 PMC8066976

[aos70032-bib-0003] Asbell, P.A. , Dualan, I. , Mindel, J. , Brocks, D. , Ahmad, M. & Epstein, S. (2005) Age‐related cataract. Lancet, 365, 599–609. 15708105 10.1016/S0140-6736(05)17911-2

[aos70032-bib-0004] Aydin, A. , Wottstein, G. , Price, L.L. , Fujimoto, J.G. & Schuman, J.S. (2003) Optical coherence tomography assessment of retinal nerve fiber layer thickness changes after glaucoma surgery. Ophthalmology, 110, 1506. 12917164 10.1016/S0161-6420(03)00493-7 PMC1939722

[aos70032-bib-0005] Bahrami, B. , Ewe, S.Y.P. , Hong, T. , Zhu, M. , Ong, G. , Luo, K. et al. (2017) Influence of retinal pathology on the reliability of macular thickness measurement: a comparison between optical coherence tomography devices. Ophthalmic Surgery, Lasers & Imaging Retina, 48, 319–325. 10.3928/23258160-20170329-06 28419397

[aos70032-bib-0007] Coassin, M. , De Maria, M. , Valentina, M. , Luca, M. , Luca, B. , Antonio, C. et al. (2019) Anterior chamber inflammation after cataract surgery: a randomized clinical trial comparing bromfenac 0.09% to dexamethasone 0.1%. Advances in Therapy, 36, 2712–2722. 31482510 10.1007/s12325-019-01076-4

[aos70032-bib-0008] Erichsen, J.H. , Forman, J.L. , Holm, L.M. & Kessel, L. (2021) Effect of anti‐inflammatory regimen on early postoperative inflammation after cataract surgery. Journal of Cataract and Refractive Surgery, 47, 323–330. 33086290 10.1097/j.jcrs.0000000000000455

[aos70032-bib-0009] Flach, A.J. (1998) The incidence, pathogenesis and treatment of cystoid macular edema following cataract surgery. Transactions of the American Ophthalmological Society, 96, 557–634. 10360304 PMC1298410

[aos70032-bib-0006] GBD 2019 Blindness and Vision Impairment Collaborators & Vision Loss Expert Group of the Global Burden of Disease Study (2021) Causes of blindness and vision impairment in 2020 and trends over 30 years, and prevalence of avoidable blindness in relation to vision 2020: the right to sight: an analysis for the global burden of disease study. Lancet Global Health, 9, e144–e160.33275949

[aos70032-bib-0011] Gharbiya, M. , Cruciani, F. , Cuozzo, G. , Parisi, F. , Russo, P. & Abdolrahimzadeh, S. (2013) Macular thickness changes evaluated with spectral domain optical coherence tomography after uncomplicated phacoemulsification. Eye, 27, 605–611. 23449512 10.1038/eye.2013.28 PMC3650275

[aos70032-bib-0012] Gietzelt, C. , Koenig, L. , Adler, W. , Schaub, F. , Heindl, L.M. , Cursiefen, C. et al. (2024) A comparative study of cystoid macula edema following glaucoma drainage device surgery versus trabeculectomy. International Ophthalmology, 44, 150. 38503938 10.1007/s10792-024-03068-y PMC10950946

[aos70032-bib-0013] Guo, S. , Patel, S. , Baumrind, B. , Johnson, K. , Levinsohn, D. , Marcus, E. et al. (2015) Management of pseudophakic cystoid macular edema. Survey of Ophthalmology, 60, 123–137. 25438734 10.1016/j.survophthal.2014.08.005

[aos70032-bib-0014] Jagow, B. , Ohrloff, C. & Kohnen, T. (2007) Macular thickness after uneventful cataract surgery determined by optical coherence tomography. Graefe's Archive for Clinical and Experimental Ophthalmology, 245, 1765–1771. 10.1007/s00417-007-0605-6 17619896

[aos70032-bib-0015] Jang, J.H. , Kim, Y.C. & Shin, J.P. (2020) Correlation between macular edema recurrence and macular capillary network destruction in branch retinal vein occlusion. BMC Ophthalmology, 20, 1–11. 32831053 10.1186/s12886-020-01611-w PMC7444240

[aos70032-bib-0016] Jayaram, H. , Kolko, M. , Friedman, D.S. & Gazzard, G. (2023) Glaucoma: now and beyond. Lancet, 402, 1788–1801. 37742700 10.1016/S0140-6736(23)01289-8

[aos70032-bib-0017] Kadziauskienė, A. , Strelkauskaitė, E. , Mockevičiūtė, E. , Ašoklis, R. , Lesinskas, E. & Schmetterer, L. (2017) Changes in macular thickness after trabeculectomy with or without adjunctive 5‐fluorouracil. Acta Medica Lituanica, 24, 93–100. 28845126 10.6001/actamedica.v24i2.3489 PMC5566947

[aos70032-bib-0018] Kawai, M. , Inoue, T. , Inatani, M. , Tsuboi, N. , Shobayashi, K. , Matsukawa, A. et al. (2012) Elevated levels of monocyte chemoattractant protein‐1 in the aqueous humor after phacoemulsification. Investigative Ophthalmology & Visual Science, 53, 7951–7960. 23132797 10.1167/iovs.12-10231

[aos70032-bib-0019] Kesim, C. , Chehab, Z. & Hasanreisoglu, M. (2022) Laser flare photometry in uveitis. Saudi Journal of Ophthalmology, 36, 337–343. 36618569 10.4103/sjopt.sjopt_119_22 PMC9811932

[aos70032-bib-0010] Kohli, P. , Tripathy, K. , Patel, B.C. (2024) Macular Edema. In: StatPearls. Treasure Island (FL): StatPearls Publishing. 35015421

[aos70032-bib-0020] Mackenbrock, L.H.B. , Baur, I.D. , Łabuz, G. , Auffarth, G.U. & Khoramnia, R. (2023) Impact of phacoemulsification parameters on central retinal thickness change following cataract surgery. Diagnostics, 13, 2856. 37685394 10.3390/diagnostics13172856 PMC10487147

[aos70032-bib-0038] Malik, A. , Sadafale, A. , Gupta, Y. , & Gupta, A. (2016) A comparative study of various topical nonsteroidal anti‐inflammatory drugs to steroid drops for control of post cataract surgery inflammation. Oman Journal of Ophthalmology, 9, 150. 27843229 10.4103/0974-620X.192268 PMC5084497

[aos70032-bib-0021] Manabe, K. , Matsuoka, Y. & Tanito, M. (2020) Incidence of macular edema development after filtration surgery. Graefe's Archive for Clinical and Experimental Ophthalmology, 258, 1343–1345. 10.1007/s00417-020-04624-9 32055953

[aos70032-bib-0022] Mwanza, J.C. , Bhorade, A.M. , Sekhon, N. , McSoley, J.J. , Yoo, S.H. , Feuer, W.J. et al. (2011) Effect of cataract and its removal on signal strength and peripapillary retinal nerve fiber layer optical coherence tomography measurements. Journal of Glaucoma, 20, 37–43. 20179622 10.1097/IJG.0b013e3181ccb93b

[aos70032-bib-0023] Nam, K.T. , Yun, C. , Seo, M. , Ahn, S. & Oh, J. (2024) Comparison of retinal thickness measurements among four different optical coherence tomography devices. Scientific Reports, 14, 3560. 38347154 10.1038/s41598-024-54109-6 PMC10861495

[aos70032-bib-0024] Nilforushan, N. , Loni, S. , Abdolalizadeh, P. , Miraftabi, A. , Banifatemi, M. , Rakhshan, R. et al. (2022) Early macular thickness changes after trabeculectomy and combined phaco‐trabeculectomy. Journal of Current Ophthalmology, 34, 160–166. 36147280 10.4103/joco.joco_333_21 PMC9487009

[aos70032-bib-0025] Ozaki, E. , Campbell, M. , Kiang, A.S. , Humphries, M. , Doyle, S.L. & Humphries, P. (2014) Inflammation in age‐related macular degeneration. Advances in Experimental Medicine and Biology, 801, 229–235. 24664703 10.1007/978-1-4614-3209-8_30

[aos70032-bib-0026] Pitale, P.M. , Chatha, U. , Patel, V. , Gupta, L. , Waisbourd, M. & Pro, M.J. (2016) Changes in macular thickness following glaucoma surgery. International Journal of Ophthalmology, 9, 1236–1237. 27588282 10.18240/ijo.2016.08.24 PMC4990593

[aos70032-bib-0027] Rao, A. & Cruz, R.D. (2022) Trabeculectomy: does it have a future? Cureus, 14, e27834. 36110452 10.7759/cureus.27834 PMC9462599

[aos70032-bib-0028] Samanta, T.K. & Saraf, P.K. (2004) Late recurrent uveitis after phacoemulsification [9] (multiple letters). Indian Journal of Ophthalmology, 52, 347. 15693340

[aos70032-bib-0029] Siriwardena, D. , Kotecha, A. , Minassian, D. , Dart, J.K.G. & Khaw, P.T. (2000) Anterior chamber flare after trabeculectomy and after phacoemulsification. British Journal of Ophthalmology, 84, 1056–1057. 10966966 10.1136/bjo.84.9.1056 PMC1723662

[aos70032-bib-0030] Tan, C.S. , Chan, J.C. , Cheong, K.X. , Ngo, W.K. & Sadda, S.R. (2015) Comparison of retinal thicknesses measured using swept‐source and spectral‐domain optical coherence tomography devices. Ophthalmic Surgery, Lasers & Imaging Retina, 46, 172–179. 10.3928/23258160-20150213-23 25707041

[aos70032-bib-0031] Tanito, M. , Hara, K. & Takai, Y. (2021) Anterior chamber flare in primary open‐angle glaucoma and exfoliation glaucoma after trabeculotomy. Graefe's Archive for Clinical and Experimental Ophthalmology, 259, 1665–1667. 10.1007/s00417-020-04962-8 33034753

[aos70032-bib-0032] Trinavarat, A. , Atchaneeyasakul, L.O. , Surachatkumtonekul, T. & Kosrirukvongs, P. (2003) Comparison of topical prednisolone acetate, ketorolac tromethamine and fluorometholone acetate in reducing inflammation after phacoemulsification. Journal of the Medical Association of Thailand, 86, 143–150. 12678152

[aos70032-bib-0033] Tugal‐Tutkun, I. & Herbort, C.P. (2010) Laser flare photometry: a noninvasive, objective, and quantitative method to measure intraocular inflammation. International Ophthalmology, 30, 453–464. 19430730 10.1007/s10792-009-9310-2

[aos70032-bib-0034] Van Velthoven, M.E.J. , Van Der Linden, M.H. , De Smet, M.D. , Faber, D.J. & Verbraak, F.D. (2006) Influence of cataract on optical coherence tomography image quality and retinal thickness. British Journal of Ophthalmology, 90, 1259–1262. 16980644 10.1136/bjo.2004.097022 PMC1857462

[aos70032-bib-0035] Verma, S. , Azad, S.V. , Takkar, B. , Temkar, S. , Chawla, R. & Venkatesh, P. (2020) Posterior segment complications following glaucoma surgeries. Indian Journal of Ophthalmology, 68, 988. 32461411 10.4103/ijo.IJO_1040_19 PMC7508102

[aos70032-bib-0036] Xiong, K. , Gong, X. , Li, W. , Yuting, L. , Meng, J. , Wang, L. et al. (2021) Comparison of macular thickness measurements using swept‐source and spectral‐domain optical coherence tomography in healthy and diabetic subjects. Current Eye Research, 46, 1567–1573. 33879001 10.1080/02713683.2021.1908566

[aos70032-bib-0037] Yilmaz, T. , Karci, A.A. , Yilmaz, İ. , Yilmaz, A. , Yildirim, Y. & Sakalar, Y.B. (2016) Long‐term changes in subfoveal choroidal thickness after cataract surgery. Medical Science Monitor, 22, 1566–1570. 27158971 10.12659/MSM.898714 PMC4918536

